# Is the place of birth related to the mother’s satisfaction with childbirth? A cross-sectional study in a rural district of the Lao People’s Democratic Republic (Lao PDR)

**DOI:** 10.1186/s12884-019-2483-4

**Published:** 2019-09-11

**Authors:** Tomomi Takayama, Khampheng Phongluxa, Daisuke Nonaka, Chika Sato, Ernesto R. Gregorio, Nouhak Inthavong, Tiengkham Pongvongsa, Sengchanh Kounnavong, Jun Kobayashi

**Affiliations:** 1 Department of Global Health, Graduate School of Health Sciences, University of the Ryukyus, 207 Uehara, Nishihara-cho, Nakagami-gun, Okinawa, Japan; 2 Lao Tropical and Public Health Institute, Samsenthai Road, Ban Kaognot, Sisattanak District, Vientiane Capital, Lao People’s Democratic Republic; 3 Asia Health and Educational Fund, 2-17-1, Nakaochiai, Shinjuku-ku, Tokyo, Japan; 4 Department of Health Promotion and Education, College of Public Health, University of the Philippines Manila/SEAMEO-TROPMED Regional Center for Public Health, Hospital Administration, Environmental and Occupational Health, 625 Pedro Gil Street, Ermita, Manila, Philippines; 5 Savannakhet Provincial Health Department, Thahea village, Kaysone-Phomvihan District, Savannakhet, Lao People’s Democratic Republic

**Keywords:** Childbirth satisfaction, Place of birth, Facility-based birth, Laos, Maternal health

## Abstract

**Background:**

The place of birth has been rapidly changing from home to health facility in Lao People’s Democratic Republic (Lao PDR) following the strategy to improve the maternal and neonatal mortality. This change in the place of birth might affect the mother’s satisfaction with childbirth. The objective of this study was to assess whether the place of birth is related to the mother’s satisfaction with childbirth in a rural district of the Lao PDR.

**Methods:**

A community-based survey was implemented in 21 randomly selected hamlets in Xepon district, Savannakhet province, between February and March, 2016. Questionnaire-based interviews were conducted with mothers who experienced a normal vaginal birth in the past 2 years. Satisfaction with childbirth was measured by the Satisfaction with Childbirth Experience Questionnaire. Using the median, the outcome variable was dichotomized into “high satisfaction group” and “low satisfaction group”. Logistic regression was performed to assess the association between place of birth and satisfaction with childbirth. Three models were examined: In Model 1, only the predictor of interest (i.e., place of birth) was included. In Model 2, the predictor of interest and the obstetrical predictors were included. In Model 3, in addition to these predictors, socio-demographic and economic predictors were included. A mixed-effects model was used to account for the hierarchical structure.

**Results:**

Among the 226 mothers who were included in data analysis, 60.2% gave birth at the health facility and the remaining 39.8% gave birth at home. Logistic regression analysis showed that the mothers who gave birth at the health facility were significantly more likely to have a higher level of satisfaction compared to the mothers who gave birth at home (crude odds ratio: 5.44, 95% confidence interval: 3.03 to 9.75). This association remained even after adjusting for other predictors (adjusted odds ratio: 6.05, 95% confidence interval: 2.81 to 13.03).

**Conclusion:**

Facility-based birth was significantly associated with a higher level of satisfaction with childbirth among the mothers in the study district where maternal and neonatal mortalities are relatively high. The findings of the present study support the promotion of facility-based birth in a rural district of the Lao PDR.

## Background

Satisfaction with childbirth has a powerful effect on the mother’s health and her baby’s well-being. High satisfaction with childbirth can enhance the mother’s relationship with their babies [1], and can empower personal strength and personal growth during the transition to motherhood [2, 3]. In contrast, poor satisfaction with childbirth is known to be associated with poorer postnatal psychological adjustment [4], a higher rate of future abortions [5], preference for a future cesarean section [6, 7], and negative feelings towards the infant and breastfeeding [8]. Hence, improving mothers’ satisfaction with childbirth is important for the well-being of mothers and their children.

Based on Subjective Well-being Theory, satisfaction with childbirth is conceptualized as a cognitive evaluation of whether the birth experience matches the mother’s personal preferences [9, 10]. Particularly, satisfaction is perceived with a “fit” between a mother’s preferences and her birth environment [11]. A review summarized 54 studies in developing countries and identified the following determinants of mother’s satisfaction with childbirth: 1) structure, such as childbirth environment; 2) process on how care was received, 3) outcome, such as child’s health condition; and 4) physical condition, such as cost and access [12].

While home-based birth is still common among rural areas in developing countries [13–15], facility-based birth has gradually increased in recent years. According to the United Nations Children’s Fund (UNICEF), the proportion of facility-based birth in the least developed countries has increased from 32% in 2009 to 54% in 2016 [14, 15]. This change is based on the World Health Organization’s (WHO) strategy [16] to prevent maternal death caused by delayed access to medical care [17]. Many developing countries are promoting facility-based birth following this strategy. For example, Lao People’s Democratic Republic (Lao PDR), one of the least developed countries in Southeast Asia, has promoted facility-based birth as a means of improving maternal and child health. Consequently, the proportion of facility-based birth in the country increased from 17% in 2005 to 62.9% in 2017 [18].

The place of birth is an important component for childbirth because it is deeply related to the physical, emotional, cultural, and social aspects of childbirth [19]. Therefore, the shift in the place of birth in Lao PDR might affect the satisfaction with childbirth of mothers. Studies that examined the association between the place of birth and mothers’ satisfaction with childbirth in the Netherlands and Canada showed that the mothers who gave birth at home were more satisfied than the mothers who gave birth at the hospital [20, 21]. In these studies, feelings of control, amount of immediate contact with the baby, and non-medical home context were identified as the reasons behind the high satisfaction with childbirth at home. However, another study has reported that Syrian women prefer hospital birth, and the main reason for the preference is the feeling of being safe [22]. Moreover, another study which was conducted in Ethiopia described that mothers who had plans to birth at a health facility were positively associated with satisfaction with childbirth [23].

Previous studies conducted in Lao PDR investigated mother’s satisfaction with delivery care and satisfaction with services provided to women who gave birth at a health facility [24, 25]. These studies described that most of the mothers who gave birth at a health facility were generally satisfied, except with the accessibility to health facilities and cleanliness. Other Lao studies have qualitatively explored why Lao women choose to give birth at home. The reasons identified were convenience, low birth costs, comfort during childbirth, and traditional practices [26, 27].

Studies comparing the satisfaction with childbirth in terms of place of birth are rare, particularly in developing countries. Therefore, this study aims to assess whether the place of birth is related to the mother’s satisfaction with childbirth in a rural district of the Lao PDR.

## Methods

### Study site and participants

We conducted a cross-sectional study in Xepon district, Savannakhet province. Xepon district is a rural district that is located along the Vietnamese border, approximately 500 km from the Vientiane capital. According to the Xepon district health office, 75% of the residents belong to ethnic minorities, such as Tri and Makong ethnic groups. The present study selected Xepon district as a poor and rural district of Lao PDR [28]. Moreover, the researchers have established a good working relationship with the local collaborators.

Xepon district has one district hospital and 14 health centers; all are public healthcare facilities. Xepon district includes 235 hamlets (a hamlet is a unit smaller than a village) according to the classification of the Xepon district health office. The district hospital covers 37 hamlets, and each health center covers between 6 and 43 hamlets.

**Fig. 1:**
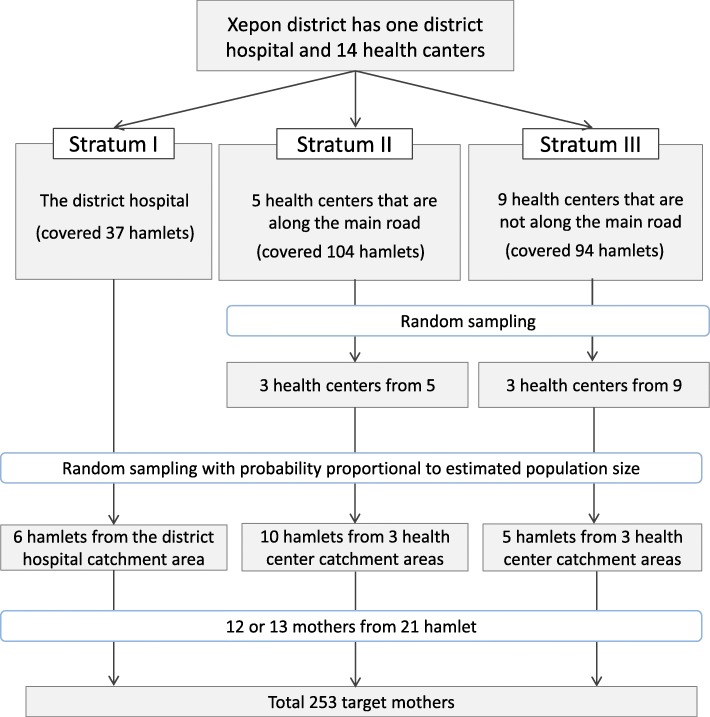
Flowchart to select the the study participants

The present study used a stratified cluster sampling to select the study participants. First, the public healthcare facilities in Xepon district were stratified into three strata: Stratum I, which is represented by the district hospital; Stratum II, which is consist of a total of five health centers located along the main road; and Stratum III, which is composed of a total of nine health centers that are not along the main road (Fig. 1). Second, we randomly selected three health centers from Stratum II and III. Third, we also randomly selected six hamlets from Stratum I, ten hamlets from Stratum II, and five hamlets from Stratum III, with probability proportional to estimated population size. Finally, we included approximately 12 study participants from each of the selected 21 hamlets. The inclusion criteria are as follows: mothers who had a singleton and had a vaginal birth within the past 2 years. The exclusion criteria were mothers who experienced infant death during the most recent birth, had a history of mental disorders, or experienced a medical intervention (e.g., forceps delivery and vacuum extraction). Additionally, the mothers whose baby had serious complications, such as congenital diseases and cerebral palsy, were excluded because of the possible influence of such complications to mothers’ satisfaction with childbirth.

In each hamlet, surveyors continued to recruit mothers until the target number of participating mothers (i.e., 12) had been reached. In one hamlet where the number of the eligible mothers who gathered did not reach the target number, the surveyors met the target number by inviting eligible mothers from a neighboring hamlet. Ultimately, 253 mothers participated in the study. We excluded six mothers based on the exclusion criteria (five mothers experienced mental disorder, and one mother experienced medical intervention). We excluded 18 mothers with missing information in the questionnaire. Three mothers who referred from home to hospital during birth were excluded because the transfer to facility during labour was likely to confound results relating to maternal satisfaction with childbirth [29]. This resulted in 226 mothers who were included in the data analysis.

### Data collection

A public health research expert (KP) trained the surveyors on the ethical issues and data collection method for 2 days. The surveyors, who were health workers from the Xepon district health office and the Savannakhet provincial health office, collected data between February and March 2016. They conducted interviews with study participants in the selected hamlets, using a semi-structured questionnaire. A day prior to the scheduled visit to the selected hamlets, the surveyors requested eligible and willing mothers through the health volunteers of the hamlets to gather at a designated place of the hamlet to participate in the study. The surveyors then visited the hamlets and interviewed the mothers who were gathered. When the surveyors had difficulty in communicating with participating mothers in the Lao language, they asked the health volunteers of the hamlet to translate the Lao language into the ethnic language.

### Variables and measurement

#### Outcome variable

The satisfaction with childbirth of mothers was measured using the seven question items obtained from the Satisfaction with Childbirth Experience Questionnaire (SWCh) [10]. SWCh is a theoretical measure based on subjective well-being theory. It was designed to assess global satisfaction with the childbirth experience and was confirmed to have content, criterion, and construct validity. High internal consistency (Cronbach’s alpha = 0.92) has been confirmed in an urban setting of the USA [10, 30]. The original SWCh consists of seven items with seven-point Likert-type response options ranging from strongly disagree to strongly agree. For each positively-phrased item, a score is obtained by giving one point for strongly disagree, and seven points for strongly agree. The code was reversed for item number 3, which is a negatively-phrased statement. The total score ranges from 7 to 49, where higher scores indicate higher satisfaction.

To validate the SWCh for use in a rural Lao setting, a native Lao speaker who is engaged in public health research did the forward translation while a native English speaker who is an expert in maternal and child health and understands the Lao language, conducted the back translation. The research team compared the original and the back-translated English versions to ensure that semantic equivalence has been maintained and that words are appropriate for rural mothers. The questionnaire translated to ethnic language involving SWCh was not prepared for this study because the ethnic groups of the study participants do not have written language system [31]. The research team pre-tested the questionnaire with 20 mothers in two rural villages of the Lao PDR. Based on the pre-test, the research team found that some respondents had difficulty understanding the Likert-type response style so that the research team developed a visual aid (additional file) and used it during the data collection to help with respondents’ understanding. The present study collected data using SWCh’s seven items. However, two items were excluded during the data analysis because inclusion of these two items resulted to the violation of the original single factor model and the Cronbach’s alpha was reduced (i.e., 0.57).

As a result of excluding the two items, the present study used the following five items: (1) In most ways, my childbirth experience was close to my ideal; (2) The conditions of my childbirth experience were excellent; (3) If I could do it over, I would change some things about my childbirth experience; (4) I am satisfied with the experience of my baby’s birth; (5) I got what I wanted out of my childbirth experience. The following two items were excluded during the analysis; My baby’s birth did not go the way I wanted it to go; If I could do it over, I would change almost nothing about my childbirth experience. In the five-item scale, the total score ranged from 5 to 35. The factor loadings of each item were above 0.5 (Table 1), and the Cronbach’s alpha was 0.75.

**Table 1:** Descriptive statistics and maximum likelihood confirmatory factor analysis factor loadings for the satisfaction with childbirth items (*n* = 226)

Items	Mean	Standard deviation	Factor I loadings
1. In most ways, my childbirth experience was close to my ideal.	5.8	1.7	0.66
2. The conditions of my childbirth experience were excellent.	6.0	1.5	0.78
3^a^. If I could do it over, I would change some things about my childbirth experience.	4.0	2.6	0.67
4. I am satisfied with the experience of my baby’s birth.	6.1	1.5	0.77
5. I got what I wanted out of my childbirth experience.	5.8	1.5	0.77

^a^Reverse scored item

#### Predictor variables

The predictor variable of interest was the place of birth for the most recent birth episode, which was measured by the question “Where did you give birth to your youngest child?”. This question has six response options such as home, health center, district hospital, provincial hospital, forest and other.

Based on previous studies which examined the predictors of childbirth satisfaction [12, 32–34], the following predictor variables were also included in the present study; parity (primiparas/multiparas), labor length (less 6 h/6 h to less than 24 h/24 h or more), pregnancy complication (yes/no), number of antenatal care received, age, educational attainment (no formal education/primary/secondary or above), ethnicity (Makong/Tri/Phouthay), possession of any vehicles (yes/no), decision maker of birth place (myself/husband/mother/mother-in-law/other), and household wealth. To assess the household wealth, a household wealth index was built by principal component analysis [35]. The information used for the wealth index was the possession of household assets (bicycle, motorcycle, two-wheel-tractor with trailer/*Tok Tok*, radio, television, mobile phone, and cow) and house types (floor/wall/roof). By the wealth index, households were ranked and divided into quartiles.

### Statistical analysis

Frequencies and medians with interquartile ranges were computed to summarize the data. The outcome variable was measured continuously. However, the present study did not use linear regression for multivariate analysis because the data did not meet the assumption for using linear regression: the residuals were not normally distributed. Therefore, the outcome variable was dichotomized into “high satisfaction group” (28 points or above) and “low satisfaction group” (fewer than 28 points), using the median (28 points) for logistic regression. Bivariate analyses were performed to assess the association between the outcome variable and each of the predictor variables using Fisher’s exact test.

Multi-level modeling was used to account for the hierarchical structure of the data: mothers (at level 1) are nested within hamlets (at level 2), and the hamlets are nested within health center catchment areas (at level 3). Thus, a three-level, mixed-effects logistic regression model was used for the multivariate analysis. Three models were examined: In Model 1, only the predictor of interest (i.e., place of delivery) was included. In Model 2, the predictor of interest and the obstetrical predictors were included. In Model 3, in addition to these predictors, socio-demographic and economic predictors were included. Stata 14.1 was used for performing the mixed-effects logistic regression analysis. Significance level was set at < 0.05 for all tests.

Additional analysis was performed to confirm if the association between place of birth and satisfaction with childbirth would change after adding two items that had been excluded in the present study analysis.

## Results

### Socio-demographic and economic characteristics of study participants

Nearly three-quarters (73.0%) were aged between 20 and 34 years old, with a median age of 25 year old (Table 2). The majority (69.5%) belong to Makong/Tri ethnic group. Most of the participants (91.2%) were farmers. More than half of the participants (65.9%) did not receive a formal education. Approximately half of the participants (50.2%) lived within 5 km of the health center, while 16.4% lived more than 9 km away. Almost three-fourths (73.8%) possessed a vehicle, such as a motorcycle or *Tok Tok* (two-wheel-tractor with trailer).

**Table 2:** Socio-demographic and economic characteristics of the study participants (*n* = 226)

Characteristics	Number	Percent
Age		
< 20 year	31	13.7
20 to 34 year	165	73.0
≥ 35 year	30	13.3
Ethnicity		
Makong/Tri	157	69.5
Phouthay	69	30.5
Occupation		
Farmer	206	91.2
Other	20	8.8
Husband’s occupation		
Famer	194	85.8
Other	32	14.2
Educational attainment		
No formal education	149	65.9
Primary (1 to 5 years)	51	22.6
Secondary or above	26	11.5
Distance from home to health facility^a^		
< 5 km	113	50.2
5 to 9 km	75	33.3
> 9 km	37	16.4
Possession of any vehicles		
Yes	167	73.9
No	59	26.1

^a^ *n* = 225

### Obstetrical characteristics of study participants

Regarding parity, three-fourths (77.4%) of the participants were multiparas (Table 3). More than half (60.2%) gave birth at a health facility, and the rest (39.8%) gave birth at home. In the breakdown of the birth place of health facility, more than half (56.6%) were gave birth at a health center, and the rest (43.4%) were gave birth at the district hospital. All 136 participants who gave birth at a health facility were attended by a skilled birth attendant. Most of the participants who gave birth at home were attended by her family member including her husband, mother, or by a traditional birth attendant: eight mothers gave birth alone. Most of the participants (77.4%) decided the place of birth by themselves. The most common self-reported health status during the pregnancy was “good” (73.9%), followed by “bad” (26.1%). The most commonly reported frequency of antenatal care visit for the pregnancy was “four times or more” (51.3%), followed by “one to three times” (25.7%) and “none” (23.0%). Few participants (6.2%) had pregnancy complications.

**Table 3:** Obstetrical characteristics of the study participants (*n* = 226)

Characteristics	Number	Percent
Parity		
Primiparas	51	22.6
Multiparas	175	77.4
Experience of neonatal death		
Yes	42	18.6
No	184	81.4
Place of birth		
Health facility	136	60.2
Home	90	39.8
Attendant of birth		
Skilled birth attendant	136	60.2
Husband	40	17.7
Mother	26	11.5
No attendant	8	3.5
Traditional birth attendant	4	1.8
Other	12	5.3
Length of Labor		
< 6 h	133	58.8
6 to 24 h	75	33.2
> 24 h	18	8.0
Decision maker of birth place		
Herself	175	77.4
Husband	24	10.6
Other	27	11.9
Chronic illness		
Yes	21	9.3
No	205	90.7
Self-reported health status during pregnancy		
Good	167	73.9
Bad	59	26.1
Antenatal care visit		
None	52	23.0
1 to 3 times	58	25.7
≥ 4 times	116	51.3
Pregnancy complication		
Present	14	6.2
Absent	212	93.8

### Bivariate association between satisfaction with childbirth and socio-demographic and economic characteristics of study participants

No socio-demographic and economic characteristics were significantly associated with satisfaction with childbirth (Table 4).

**Table 4:** Bivariate association between satisfaction with childbirth score and socio-demographic and economic characteristics of the study participants (*n* = 226)

Characteristics	Higher score group		Lower score group		*p* value^a^
	Number	Percent	Number	Percent	
Age					
< 20 year	14	45.2	17	54.8	0.398
20 to 34 year	90	54.5	75	45.5	
≥ 35 year	13	43.3	17	56.7	
Ethnicity					
Makong/Tri	41	59.4	28	40.6	0.149
Phouthay	76	48.4	81	51.6	
Religion					
Buddhism	41	59.4	28	40.6	0.257
Animism	74	49.0	77	51.0	
Other	2	33.3	4	66.7	
Occupation					
Farmer	109	52.9	97	47.1	0.350
Other	8	40.0	12	60.0	
Husband’s occupation					
Farmer	102	52.6	92	47.4	0.572
Other	15	46.9	17	53.1	
Educational attainment					
No formal education	73	49.0	77	51.0	0.549
Primary (1 to 5 years)	29	56.9	22	43.1	
Secondary or above	15	57.7	11	42.3	
Distance from home to health facility^b^					
< 5 km	60	53.1	53	46.9	0.881
5 to 9 km	37	49.3	38	50.7	
> 9 km	19	51.4	18	48.6	
Possession of any vehicles					
Yes	92	55.1	75	44.9	0.098
No	25	42.4	34	57.6	
Household wealth quartile					
First (poorest)	34	59.6	23	40.4	0.347
Second	31	55.4	25	44.6	
Third	26	47.3	29	52.7	
Fourth (least poorest)	26	44.8	32	55.2	

^a^Fisher’s exact test

^b^*n* = 225

### Bivariate association between satisfaction with childbirth and obstetric characteristics of study participants

The participants who gave birth at a health facility were significantly more likely to have a higher level of satisfaction with childbirth (Table 5) compared to the participants who gave birth at home (*p* < 0.001). Being the decision maker of the birth place was significantly associated with satisfaction with childbirth (*p* < 0.001). Receiving antenatal care was significantly associated with satisfaction with childbirth (*p* = 0.035). The participants who did not have pregnancy complications were significantly more likely to have a higher level of satisfaction with childbirth compared to the participants who had pregnancy complications (*p* = 0.026).

**Table 5:** Bivariate association between satisfaction with childbirth score and obstetrical characteristics of the study participants (*n* = 226)

Characteristics	Higher score group		Lower score group		*p* value^a^
	Number	Percent	Number	Percent	
Parity					
Primiparas	24	47.1	27	52.9	0.525
Multiparas	93	53.1	82	46.9	
Experience of neonatal death					
Yes	21	50.0	21	50.0	0.865
No	96	52.2	88	47.8	
Place of birth					
Health facility	92	67.6	44	32.4	< 0.001
Home	25	27.8	65	72.2	
Length of labor					
< 6 h	66	49.6	67	50.4	0.770
6 to 24 h	41	54.7	34	45.3	
> 24 h	10	55.6	8	44.4	
Decision maker of birth place					
Herself	78	44.6	97	55.4	< 0.001
Husband	19	79.2	5	20.8	
Other	20	74.1	7	25.9	
Chronic illness					
Present	8	38.1	13	61.9	0.252
Absent	109	53.2	96	46.8	
Self-reported health states during pregnancy					
Good	89	53.3	78	46.7	0.453
Bad	28	47.5	31	52.5	
Antenatal care visit					
None	20	38.5	32	61.5	0.035
1 to 3 times	27	46.6	31	53.4	
≥ 4 times	70	60.3	46	39.7	
Pregnancy complication					
Present	3	21.4	11	78.6	0.026
Absent	114	53.8	98	46.2	

^a^Fisher’s exact test

### Multivariate analysis for the association between satisfaction with childbirth and place of birth

Logistic regression analysis showed that mothers who gave birth at a health facility were significantly more likely to have a higher level of satisfaction compared to the mothers who gave birth at home (crude odds ratio: 5.44. 95% confidence interval: 3.03 to 9.75) in Model 1 (Table 6). In Model 2, the association between the place of birth and satisfaction with childbirth remained statistically significant (adjusted odds ratio: 7.30, 95% confidence interval: 3.51 to 15.16). In Model 3, the association remained statistically significant (adjusted odds ratio: 6.05, 95% confidence interval: 2.81 to 13.03). Apart from the association with place of birth, pregnancy complication (adjusted odds ratio: 0.08, 95% confidence interval: 0.02 to 0.42) and family member’s decision on birth place (adjusted odds ratio: 3.80, 95% confidence interval: 1.62 to 8.92) were also significantly associated with childbirth satisfaction in Model 3.

**Table 6:** Multivariate analysis for the place of birth and satisfaction with childbirth score after adjusting for confounding variables (*n* = 226)

	Model 1		Model 2		Model 3	
	OR^a^	95% CI^b^	AOR^c^	95% CI^b^	AOR^c^	95% CI^b^
Place of birth						
Home	1.00	Reference	1.00	Reference	1.00	Reference
Health facility	5.44	3.03 to 9.75	7.30	3.51 to 15.16	6.05	2.81 to 13.03
Parity						
Primiparas			1.00	Reference	1.00	Reference
Multiparas			2.51	1.09 to 5.79	2.30	0.95 to 5.58
Labor length						
< 6 h			1.00	Reference	1.00	Reference
6 to 24 h			1.15	0.60 to 2.18	1.32	0.68 to 2.59
> 24 h			0.94	0.31 to 2.83	0.75	0.23 to 2.48
Pregnancy complication						
Absent			1.00	Reference	1.00	Reference
Present			0.15	0.37 to 0.65	0.08	0.02 to 0.42
Received antenatal care						
None			1.00	Reference	1.00	Reference
1 to 3 times			0.61	0.24 to 1.54	0.51	0.20 to 1.33
≥ 4 times			1.03	0.44 to 2.40	1.04	0.41 to 2.63
Age						
< 20 year					1.00	Reference
20 to 34 year					1.19	0.42 to 3.39
≥ 35 year					0.94	0.24 to 3.77
Educational attainment						
No formal education					1.00	Reference
Primary school					0.94	0.41 to 2.14
Secondary or above					0.65	0.20 to 2.13
Ethnicity						
Makong/Tri					1.00	Reference
Phouthay					0.81	0.34 to 1.92
Household wealth quartile						
First (poorest)					1.00	Reference
Second					1.40	0.55 to 3.57
Third					1.37	0.49 to 3.80
Fourth (least poor)					1.07	0.38 to 3.01
Possession of any vehicles						
Yes					1.00	Reference
No					0.88	0.41 to 1.91
Decision maker of birth place						
Herself					1.00	Reference
Other					3.80	1.62 to 8.92

^a^Odds ratio. ^b^Confidence Interval. ^c^Adjusted odds ratio

The additional analysis using SWCh’s seven original items also showed that mothers who gave birth at a health facility were significantly more likely to have higher level of satisfaction compared to the mothers who gave birth at home (adjusted odds ratio: 7.63, 95% confidence interval: 3.25 to 17.91 in Model 3) (Table 7).

**Table 7:** Multivariate analysis for the place of birth and satisfaction with childbirth score after adjusting for confounding variables (SWCh 7item version) (*n* = 226)

	Model 1		Model 2		Model 3	
	OR^a^	95% CI^b^	AOR^c^	95% CI^b^	AOR^c^	95% CI^b^
Place of birth						
Home	1.00	Reference	1.00	Reference	1.00	Reference
Health facility	5.17	2.66 to 10.02	8.83	3.91 to 19.95	7.63	3.25 to 17.91
Parity						
Primiparas			1.00	Reference	1.00	Reference
Multiparas			3.13	1.25 to 7.81	3.22	1.23 to 8.43
Labor length						
< 6 h			1.00	Reference	1.00	Reference
6 to 24 h			1.34	0.66 to 2.72	1.37	0.66 to 2.81
> 24 h			0.39	0.12 to 1.27	0.36	0.11 to 1.21
Pregnancy complication						
Absent			1.00	Reference	1.00	Reference
Present			0.24	0.06 to 0.97	0.23	0.05 to 1.03
Received antenatal care						
None			1.00	Reference	1.00	Reference
1 to 3 times			0.35	0.13 to 0.94	0.30	0.11 to 0.86
≥ 4 times			0.58	0.23 to 1.50	0.47	0.17 to 1.31
Age						
< 20 year					1.00	Reference
20 to 34 year					0.51	0.16 to 1.65
≥ 35 year					0.41	0.09 to 1.85
Educational attainment						
No formal education					1.00	Reference
Primary school					1.29	0.53 to 3.13
Secondary or above					0.84	0.22 to 3.16
Ethnicity						
Makong/Tri					1.00	Reference
Phouthay					0.68	0.24 to 1.90
Household wealth quartile						
First (poorest)					1.00	Reference
Second					0.63	0.22 to 1.76
Third					0.72	0.24 to 2.21
Fourth (least poor)					0.83	0.26 to 2.62
Possession of any vehicles						
Yes					1.00	Reference
No					0.78	0.33 to 1.88
Decision maker of birth place						
Herself					1.00	Reference
Other					1.78	0.74 to 4.28

^a^Odds ratio. ^b^Confidence Interval. ^c^Adjusted odds ratio

## Discussion

The present study showed that mothers who gave birth at the health facility were significantly more likely to have a higher level of satisfaction with childbirth compared to mothers who gave birth at home in a rural district of Lao PDR. The association between the place of birth and satisfaction with childbirth can be considered robust because the strength of the association was strong (odds ratio: 5.44) [36] and remained largely unchanged (adjusted odds ratio: 6.05) even after adjusting for potential confounding factors.

One possible reason for the higher satisfaction among women who gave birth at a health facility is their exposure to delivery-related policy and programs, such as ‘Free Maternal Health Service Policy’ [37] and ‘Skilled Birth Attendance Development Plan’ [38], which are implemented at Lao health facilities. The ‘Free Maternal Health Service Policy’ was introduced by the Lao government in 2012 to promote the utilization of maternal health service and reducing financial burden. This policy covers all treatment, transportation support (2.5 to 6.5 USD) and a food fee during their stay in health facilities [37]. Because financial burden has been identified as a factor that influences mothers’ satisfaction with childbirth [12], the implementation of this policy could have contributed to their satisfaction. Moreover, ‘Skilled Birth Attendance Development Plan’ might have also contributed to improved maternal and child health service and eventual mothers’ satisfaction [39]. This plan aims to ensure that adequate human resources are recruited, retained, supervised, and provided with the necessary skills and complemented by enabling environment (e.g., sphygmomanometer and fetoscope).

Our main finding concurs with several studies conducted in Lao PDR and other developing countries. Two studies conducted in Xiengkhuang province and Oudomxay province described that more than 80% of mothers who gave birth at the hospital were generally satisfied with the maternity health care, especially with the attitude of the medical staffs and medical service of the facilities [24, 25]. Other studies that focused on satisfaction with childbirth in Sri Lanka, Nepal, and Ethiopia also described that many mothers were satisfied with the health care services provided by a health facility, except for access, cost and waiting time in the hospital [40–43].

The low satisfaction of mothers who gave birth at home in the current study might have been influenced by the lack of assistance provided by skilled birth attendants. In the present study, no mothers who gave birth at home were attended by skilled birth attendants, and only 1.8% of them were supported by traditional birth attendants. Many of them were supported by a husband or family members. Skilled birth attendants give appropriate advice, guidance, and support during pregnancy, childbirth, and postnatal period [44]. Assistance from professionals could give a sense of security to mothers during labor. According to previous studies, the sense of security is described as one of the essential components of satisfaction with childbirth [30, 45].

The main findings of the present study are not consistent with the primary findings of similar studies conducted in developed countries. Those studies described that mothers who gave birth at home were more satisfied than the mothers who gave birth at the hospital, and reported that less intervention and having close contact with the baby contributed to increasing the childbirth satisfaction [20, 21, 46, 47]. This difference might be attributed to the high quality of health services available in developed countries. Home-based delivery in a developed country is usually assisted by the skilled birth attendant, and if an abnormality occurs during birth, mothers can be immediately transferred to a medical facility under the care of experts.

In the present study, other factors that were positively related to satisfaction with childbirth were: 1) absence of pregnancy complication, and 2) family members’ decisions on birth place. However, the present study could not identify the association because this study did not adjust for these confounding variables. To enhance the mother’s satisfaction with childbirth in developing countries, future studies are recommended to explore the factors that are associated with the satisfaction with childbirth in consideration of high maternal and infant mortality and morbidity.

The utility rates of maternal healthcare services found in the present study were similar to those reported in the Lao Social Indicator Survey conducted between 2016 and 2017 [18]. For example, the proportion of facility-based birth was 60.2% in the present study, whereas it was 60.5% in Savannakhet province. Similarly, the proportion of mothers who availed of antenatal care more than four times was 51.3% in the present study, whereas it was 53.4% in Savannakhet province. Therefore, the findings of the present study might be apply to other rural districts in Savannakhet province.

To the best of our knowledge, the present study is the first to quantitatively study the association between place of delivery and satisfaction with childbirth in Lao PDR or any other Southeast Asian country. Previous studies in Lao PDR have explored the satisfaction with maternal care services in health facilities. This study is original in that it includes hypothesis-based analysis, including not only the mothers who gave birth at a health facility but also the mothers who gave birth at home. The Lao Government’s strategy of promoting facility-based birth might have contributed to the mother’s health and her baby’s well-being, as well as safe birth. The results of this study support the strategy of promoting facility-based birth in Lao PDR.

Our study has some limitations. First, the study included mothers who arrived first in the meeting place of their hamlet. This group of mothers might have more interest in healthcare compared to mothers who arrived late. This possible selection bias might have contributed to higher satisfaction among mothers who gave birth at a health facility. Second, the present study used the five-item scale that has not been used in previous studies. The researcher had to eliminate the two items from the original, seven-item SWCh in order to keep the original single factor model and to increase the reliability of the Cronbach’s alpha. However, the five-item scale used in the present study ensured construct validity; the main findings are unlikely to change even if the original seven-item scale had been used in the present study. Third, while the present study found significant association between the place of birth and satisfaction with childbirth, due to the cross-sectional nature of the study, this association does not necessarily mean causation. The fourth limitation is that the present study did not measure the satisfaction of maternal care. Therefore, the present study could not discuss the quality of maternal care provided by health facilities in Lao PDR. To promote facility-based birth and enhance the satisfaction with childbirth, future studies will be needed to evaluate the quality of maternal care and supply-side readiness of facility-based birth [37, 48, 49].

## Conclusion

The facility-based birth was significantly associated with a higher level of satisfaction with childbirth among mothers in the study district where maternal and neonatal mortalities are relatively high. There is a possibility that women living in a rural district of Savannakhet province can improve their satisfaction with childbirth if they change the place of birth from home to a facility. The results of the present study support the strategy of promoting facility-based birth in Lao PDR.

## Data Availability

The datasets used and/or analyzed in this study are available from the corresponding author on reasonable request.

## Abbreviations

Lao PDR: Lao People’s Democratic Republic
SWCh: Satisfaction with Childbirth Experience Questionnaire
UNICEF: United Nations Children’s Fund
WHO: World Health Organization
